# Supplementary material for: Are carbon emissions trading and green financial instruments synergistic? -Comprehensive quantitative research based on content analysis
Source: PLoS One. 2024 Mar 7;19(3):e0298601. doi: 10.1371/journal.pone.0298601 (PMC10919601; doi:10.1371/journal.pone.0298601)
Supplement: S1 Data — (ZIP) [file pone.0298601.s001.zip › Data/地级市Z_F工作报告.zip!/╣π╢1⁄2/╜¡├┼╩╨/└┤╫╘╣1⁄2╓┌║┼í╛┬φ┐╦╩2╛▌═°í┐_2013.docx]

各位代表：

　　我代表市人民政府向大会作政府工作报告，请予审议，

　　并请政协各位委员和其他列席人员提出意见。

　　2012年工作回顾

　　2012年江门市主要经济指标预计完成情况表

　　指 标　　　绝对值　 增速（%）

　　生产总值　　　　1910亿元　　8.1

　　地方公共财政预算收入 135亿元　　13.3

　　全社会固定资产投资　　850亿元　　14.6

　　社会消费品零售总额　　807亿元　　10

　　外贸进出口总额　　188亿美元　 6.1

　　2012年是我市真抓实干迎挑战、抢抓机遇促发展的一年。面对严峻的经济形势，市政府在省委、省政府和市委的正确领导下，在市人大及其常委会和市政协的监督支持下，大力推进“种树、搭桥修路、抓大项目”三件大事，把“三促一帮”[1]作为经济工作的主抓手，实施《珠三角规划纲要》工作连续第二年在全省考评中获评优秀，顺利通过全国文明城市首次复检，基本实现了稳增长、调结构、惠民生的预期目标任务。

　　一、扎实推进“三促一帮”，转型升级迈出新步伐

　　全市各级财政投入2.74亿元，争取上级资金3.38亿元，大力推进“三促一帮”各项政策措施的落实。各级领导班子积极带头招项目、抓落实，大力开展“暖企”活动，通过深入基层调研、现场办公会、督办会等形式切实协调解决制约基层和企业发展的问题。各级人大、政协紧紧围绕市的中心工作积极开展视察调研、检查督办工作。通过积极应对、主动作为，我市经济运行呈现“前低后高”走势，据初步统计，全市生产总值完成1910亿元，各项主要经济指标实现“稳中有进”，地方公共财政预算收入增幅超过全省平均水平。

　　先进制造业发展实现新突破。形成了核心工业园、战略性新兴产业、传统产业转型升级“三轮驱动”的格局。“双转移”[2]工作被省评为优秀，台山清洁能源（核电）装备产业园纳入省产业转移园区。江沙示范园棠下片区和雅瑶片区完成统一规划。江门国家高新区以“三资融合”[3]模式启动总部科技园、科技创新创业园建设。以LED新光源、船舶工业、高端装备制造、汽车零部件产业为代表的战略性新兴产业和先进制造业加快发展，LED产业、船舶工业总产值分别增长18.2%和18%，省市共建汽车零部件产业园挂牌，省轨道交通装备产业联盟落户我市。与省海洋渔业局达成共建海洋经济强市协议，成功争取“海域使用直通车”试点，海洋产业总产值达452亿元，增长28%。大力推进“四个100”工程[4]，传统产业转型升级加快。成功创建1个国家级和2个省级外贸转型升级示范基地。着力推进188个工业技改项目实施，技改投资完成55亿元，增长38%。成功举办第六届“省长杯”工业设计大赛暨首届“五邑杯”创新工业设计大赛、市工业设计与企业对接活动周，促进“江门制造”向“江门创造”转型。扶持企业力度加大，累计减免企业社会保险费2.7亿元。发放扶持就业补贴3806.64万元，258家企业、3.28万劳动者受益。2.6万家企业、25.51万人享受流动人员调配费减免优惠。建成政企通信息服务平台和中小企业服务机构联盟，接受服务企业达2000多家。全市累计扶持个体工商户转型升级为企业超2000户。

　　服务业发展加快。全市本外币存贷比为51.85%，创近8年新高，获得各银行机构授信860亿元。金融体系不断完善，新引进汇丰、恒生、光大、招商银行落户我市。企业上市工作步伐加快，科恒实业成功上市，2家企业首发申请成功过会。江海区获全省金融稳定奖。旅游业持续较快发展，与澳门签订了旅游合作备忘录，成功举办第七届广东(江门)国际温泉旅游节、江门台山文化旅游博览会，全市旅游总收入185亿元，同比增长20%；接待游客2987万人次，同比增长12%。物流业增加值增长10.5%。电子商务加快发展，500多家企业入选广货网上行市场主体。成功举办江门（成都）名优商品展销会暨旅游产品推介会和第四届珠中江进出口商品展销会。

　　自主创新能力增强。科技创新投入力度加大，全市研究与开发经费支出占生产总值比例1.25%。新增国家级高新技术企业37家、省民营科技企业56家、省创新型企业1家。成为广东省部院产学研结合示范市。成立科技创业风险投资基金。发放了首届标准化战略资金。国家机械装备检测重点实验室加快建设，国家摩托车检验中心取得华南地区唯一的3C产品认证[5]资质，省级质量监督电声产品、水暖卫浴产品检验站分别投入使用和完成建设。发明专利申请量增长64%。

　　二、紧抓重大项目建设和引进，经济发展后劲增强

　　招商引资和增资扩产成效显著。成功举办2012年江门市招商推介会，向社会推出招商项目126个、金额超1000亿元，现场签约项目39个、合同投资金额780亿元。承办省“区洽会”暨乡贤反哺工作会议取得显著成果，申报合作项目157个，总金额407.99亿元，均居全省之首。成功引进大连机床、西门子、鸿特精密等大型项目。李锦记、大长江、富华重工、维达纸业、亚太纸业、卡夫食品、金羚电器、西铁城LED、天地壹号等相继增资扩产，全市增资扩产项目329宗，实际到位资金131.7亿元。

　　产业项目建设进展顺利。全市重点项目建设完成投资341.32亿元，投资完成率120.6%，为近年来完成率最高的一年。海信首期平板电视机项目正式投产，广东南车、台山核电、康师傅、普利司通、星辉造纸、崖门环保电镀基地等产业重大项目建设扎实推进。中国江门国际绿色光源博览交易中心建设启动。

　　交通基础设施建设加快。全市交通建设完成投资45亿元，江顺大桥完成投资5.35亿元，广珠铁路全线贯通，佛开高速扩建主线工程、东华大桥、里村大道及迎宾路里村段、新会启超大道二期、台山石花北路、鹤山大鹏路、恩平圣贵线和大合线完工通行，江门大道北线、建设路——迎宾路立交、胜利南路、开平环城公路、广中江高速、江罗高速建设扎实推进。高新区公共码头项目建设启动。325国道鹤山收费站和潮连大桥收费站取消收费。与珠三角其他城市实现年票互认。

　　城市建设快速推进。滨江新区开发建设取得突破性进展，创新采用DBO[6]模式引进保利集团参与江门体育中心建设，广东银葵综合医院项目完成供地手续。江门义乌小商品城、恩平鳌峰世纪商业广场建成营业，江门国翔时代广场、江海西江新城、新会美吉特广场、台山中环广场、开平东汇城、鹤山广场、恩平锦江国际新城等商贸中心加快建设。“三旧”[7]改造累计投入50.49亿元，完成改造总面积1689亩。甘化厂片区改造启动，江会路片区开发前期工作加快推进。

　　三、加快现代农业发展，城乡统筹发展扎实推进

　　农业产业化步伐加快。全市新增省级重点农业龙头企业4家、市级重点农业龙头企业7家、农民专业合作社63家。成功申报省农业科技园区。全市“三品一标”[8]农产品总数位居全省第三，建立新会陈皮产业园。成功举办第三届江门市农业博览会和第二届中国（江门）锦鲤博览会。成为计划总投资2亿美元的国内首个世界银行贷款农业面源污染治理项目实施的主要地区。落实政策性水稻种植保险面积41万亩。建立水稻高产示范点77个，全市粮食总产量增产5%，粮食安全得到进一步保障。

　　农村生产生活条件明显改善。全市各级财政“三农”投入57.9亿元，比上年增长20.35%。投入民生水利建设7.2亿元，“农田水利万宗工程”[9]和“双千”工程[10]建设扎实推进。有效开展正面袭击我市特大台风“韦森特”的防御和救灾复产工作，防台风取得“零伤亡”成绩。江新联围干堤加固工程被评为中国优质水利工程奖“大禹奖”。完成农村公路建设200公里。60%的村庄开展村庄整治工作。城乡电力基础设施完成投资19.4亿元。建成7个宜居城镇、14个宜居村庄和2个名镇、44个市县两级名村，台山市斗山镇成为全省首批名镇示范点。

　　农村管理水平进一步提升。建成农村社区服务中心92个。基层农村集体“三资”[11]管理机构进一步完善，70个镇（街）落实农村集体财务创新管理模式。实施农村公益事业建设一事一议财政奖补办法。“双到”扶贫[12]取得显著成绩，对省下达任务的105条贫困村累计投入帮扶资金4.04亿元，有劳动能力贫困户脱贫率达100%。

　　四、加强生态保护，宜居环境不断改善

　　开展“森林围城、树林进城”绿色行动取得明显成效，参与活动人次达60多万，全市共投入绿化建设资金1.78亿元，种植树木（苗）817万棵。完成天沙河两岸9.6公里绿化美化景观建设，蓬江篁庄、怡福、双龙、龙湾公园及新会圭峰山绿护屏、台山通济公园、开平人民公园、恩平鳌峰山公园完成升级改造，鹤山沙坪河南岸综合整治工程完工，元宝山体育公园一期工程和丰乐山公园加快建设。完成市区35个平交节点、主要道路中间绿化带、高速公路出入口的绿化升级。新建城市绿道57公里、城市绿道驿站8个。完成生态景观林带建设里程104公里，造林面积1.54万亩。建设“林业生态文明村”140个。全市建成区绿化覆盖率42.5%，绿地率40.2%，人均公园绿地面积16.8平方米。环境保护力度加大，顺利通过国家环保模范城市复检。潭江流域保护工作扎实推进，饮用水源水质保持良好。全市新增污水截污管网30公里，在建污水处理厂3座，预计新增处理能力8万吨/日，城镇生活污水处理率达到82%以上。新建生活垃圾转运站24座，全市生活垃圾无害化处理率达85%以上。建成PM2.5监测网络并向社会发布监测结果。全国首个大气复合污染超级监测站落户我市。市区全年环境空气质量符合国家二级标准，空气质量日报优良率99.5%。节能减排和资源节约集约利用工作水平提升，成为全省4个低碳试点城市之一和首个商贸酒店节能试点城市，银洲湖纸业基地被列入国家工业循环经济重大示范工程。全市完成66个减排项目。11家企业被认定为省清洁生产企业。安装LED路灯4650盏。超额完成省下达淘汰落后产能任务。节约集约用地力度加大，全市盘活存量土地2.02万亩。

　　五、加快文化名市建设，侨乡品牌进一步打响

　　成功举办第三届世界江门青年大会。入选省第四批省级非物质文化遗产名录和省第一批非物质文化遗产传承基地的项目分别达5个和2个。新会林缉光艺术博物馆、开平市谭逢敬艺术院、恩平市文化艺术中心等文化场馆投入使用。成功举办全国册页书法作品（开平）展、中国台山玉雕刻创作大赛、“南粤幸福活动周”和珠中江民歌大赛、江门市民大讲坛、侨乡阅读文化节等文化活动。新增3个省级文物保护单位，甘化厂工业遗产调查和保护工作启动。大力推进文化下乡、进社区活动，新建（改造）农村（社区）文化室438间、农家书屋190家，送戏下乡960场，农村电影放映1.33万场，成为全国百姓健康舞文化惠民示范城市。实现广播电视“村村通”[13]、“渔船通”[14]。成为全国文化体制改革先进城市，市县国有电影公司转企改制成功。文化产业加快发展，第二届江门侨乡动漫节成功举办，《我是缤果》开创我市动画片在央视播出的先河。

　　六、加大民生改善力度，群众得到更多实惠

　　全市各级财政民生投入84.9亿元，增长18.49%，省、市十件民生实事按进度完成，“创文”长效机制逐步健全。

　　保就业增收入成效显著。城镇居民人均可支配收入、农村居民人均纯收入分别比上年增长13.5%和13.3%，高于全市生产总值增幅。实施“校园对接，双招互助”[15]工程取得实效。全市城镇新增就业4.77万人，失业人员再就业3.47万人，就业困难人员再就业2671人，城镇登记失业率2.35%。全市开展农村劳动力技能培训4万人，新增转移农村劳动力2.2万人。积极搭建政校企三位一体的人力资源对接服务平台。全市新增高技能人才1.27万人。

　　社会保障体系进一步健全。全市城镇职工基本养老、基本医疗、失业、工伤、生育保险参保人数分别比上年增长9.29%、9.1%、3.27%、7%、3.63%。及时提高社保待遇，全市18.8万人参加养老金调整，人均增加143元。大力推进社会保障“一卡通”，发放社会保障卡160万张。城乡医保待遇水平进一步提高。建立了城乡一体化的居民社会养老保险制度，实现制度和参保人数全覆盖，城乡居民社会养老保险工作被评为全国先进。全征地农民基本养老保障试点工作扎实推进，试点范围不断扩大。超额完成省下达的保障性住房建设和华侨农场危房改造任务。城乡低保标准进一步提高，年人均收入低于2500元的城乡居民全部纳入低保救助范围，实现“五保”[16]供养标准不低于上年度农村居民人均纯收入的60%。在全省率先实施残疾儿童学前三年免费教育。扩大高龄老人政府津贴发放范围，建成蓬江区居家养老服务示范中心。涌现出“思利及人”公益基金、五邑慈善会、昌兴关爱基金等一批慈善机构，慈善公益万人行、“扶贫济困日”等大型慈善公益活动共筹集善款超2亿元。

　　各项社会事业加快发展。市民群众最关心最迫切解决的热点民生工作加快推进，完成10个市区农贸市场改造升级，新增市区公交车250标台，全市新建平价商店85家，新建和改建农村户厕1.1万个。教育现代化建设扎实推进，蓬江区成功创建省推进教育现代化先进区。学前教育加快发展，全市学前三年入园率达93.23%。义务教育阶段规范化学校建设达标率96%。外来务工人员子女读书问题妥善解决。农村义务教育学生营养改善计划试点工作扎实推进。成功创建1所国家中等职业教育改革发展示范学校、1所省示范性中等职业学校。全市高中阶段毛入学率达96.15%。成为全国未成年人思想道德建设工作先进城市。顺利通过国家卫生城市复审，代表国家通过世卫组织消除新生儿破伤风现场认证，新增2家三级甲等综合医院。创建省卫生镇4个、省卫生村34个、市卫生村51个。体育事业再创佳绩，在伦敦残奥会上我市体育健儿勇夺二金一银一铜。国防动员体系不断完善，获得“全国双拥模范城”五连冠。人口计生工作完成省下达目标任务。社会保障资金审计工作获得全国优秀。此外，对台、民族、宗教、人防、打私、统计、妇女儿童、档案、方志、气象、红十字会等工作进一步加强。

　　社会管理创新取得新进展。全市新增社会组织230个，为历年来增长最快的一年，其中新评估4A级社会组织[17]2个。社工、义工服务队伍不断壮大，在全省率先创建社会工作专业人才孵化培育基地，登记成立了江门市社会工作协会和10家民办社工机构。在全国首创劳资纠纷义务调解员制度。鹤山“大调解”格局成为省第一批社会创新观察试点项目。“平安江门”建设加快，新安装治安视频监控摄像头2331个、治安视频卡口106个，市公安智能交通控制中心项目完成建设。应急处置能力得到提高。“三打两建”[18]工作取得实效，全市共查处“三打”案件14556宗，查处“保护伞”185人，抓获犯罪嫌疑人10208人，“两建”试点工作扎实推进。加强产品质量和食品药品安全监管整治。安全生产隐患治理成效显著，各项安全生产事故指标同比全面下降，没有发生重大恶性事故，消防领域实现“零伤亡”受到省通报表扬。

　　七、持续加强政府建设，服务效能明显提升

　　坚持改革创新，政府执行力、协同力、公信力和创新力进一步提升，政府职能加快转变。

　　科学决策水平稳步提高。坚持依法行政，完善法治政府建设配套制度，荣获全省首批法治先进城市称号。自觉接受市人大及其常委会和市政协的监督，人大议案和建议、政协提案办理满意率分别达97.5%、100%，被省政府评为2008－2012年建议提案办理先进单位。坚持人大代表约见市长、局长和民生热线制度，建立重大行政决策专家咨询论证制度。

　　各项改革进一步深化。富县强镇、简政强镇事权改革和事业单位改革深入开展。县级大部门体制改革全面铺开，蓬江区、江海区改革试点工作完成。深化行政审批制度改革，385项行政审批事项列入第一批市级行政审批制度改革事项。在全省率先开通网上办事大厅，提供560项服务事项、1122条行政审批流程全程在线申办服务。在全省率先开通“网上申请、双向快递”企业登记注册系统。17个街道建成“一站式”政务服务中心。建成全省首个行政服务标准化研究推广基地。首次开展全市公务员标兵评选工作。严格落实倒逼机制，推行“两重”[19]责任制，实施市长办公会督办通报制度，强化飞行、暗访督查，政府执行力得到提升。加大行政投诉调处力度。落实信访维稳领导“一岗双责”和包案责任制，有效解决一批涉及群众切身利益的问题。

　　廉洁城市建设启动。从廉洁高效政府、廉洁公正司法、廉洁党员干部队伍、廉洁城市文化、廉洁诚信社会五个方面大力推进廉洁城市建设，效能监察、廉政建设和反腐败工作进一步加强。“三公”经费[20]得到严格控制，实现了“五个零增长”。建立市级公共资源交易平台，政府集中采购制度不断完善。加快行政处罚自由裁量标准的审核和公布。做好广东省县级政府绩效管理试点工作，将政府绩效管理纳入电子监察工作取得进展。

　　各位代表，上述成绩的取得，离不开上级党委、政府和市委的正确领导，离不开市人大、市政协与各民主党派的监督支持，离不开全市人民、外来投资者和建设者以及驻江门军警部队的共同努力，离不开港澳台同胞、海外侨胞以及国际友人的关心帮助。在此，我代表市人民政府，向所有参与、支持和关心江门发展建设的各界人士，表示衷心的感谢！

　　在充分肯定成绩的同时，我们也清醒地看到工作中还存在不少突出问题：经济结构不合理，实体经济不够强，抗风险能力弱；城镇化水平不高，统筹城乡发展、推进公共服务均等化的步伐仍不够快；政府服务和办事效率离市民、企业和社会各界的期望还有一定差距。这些问题，我们将高度重视，采取有力措施加以解决。

　　2013年工作安排

　　今年经济社会发展的主要预期目标

　　生产总值增长8%；

　　人均生产总值增长7%；

　　固定资产投资增长14%；

　　社会消费品零售总额增长11%；

　　外贸进出口总额增长4%；

　　地方公共财政预算收入增长11%；

　　居民消费价格涨幅控制在3.5%左右；

　　城镇居民人均可支配收入增长11%，农村居民人均纯收入增长13%；

　　城镇登记失业率控制在3.2%以内；

　　节能减排约束性指标完成省下达的目标任务。

　　今年是贯彻落实党的十八大精神、实现“十二五”规划目标的重要一年。我们要全面贯彻落实党的十八大、中央经济工作会议、习近平总书记视察广东重要讲话、胡春华书记在江门调研重要讲话和市委十二届三次全会精神，把人民对美好生活的向往作为奋斗目标，求真务实、开拓进取，着力做大做强实体经济，着力提高城镇化水平，着力优化生态环境，着力创新社会管理，着力改善民生，着力加强政府建设，努力开创幸福侨乡建设新局面。

　　今年全市经济社会发展的主要预期目标是：生产总值增长8%，人均生产总值增长7%，固定资产投资增长14%，社会消费品零售总额增长11%，外贸进出口总额增长4%，地方公共财政预算收入增长11%；居民消费价格涨幅控制在3.5%左右；城镇居民人均可支配收入增长11%，农村居民人均纯收入增长13%；城镇登记失业率控制在3.2%以内；节能减排约束性指标完成省下达的目标任务。

　　为实现上述目标，要切实做好以下工作：

　　一、做大做强实体经济

　　牢牢把握发展实体经济这一经济基础，坚持发展是硬道理，把经济发展作为解决一切问题的根本，推动经济“稳中求进”。

　　强化重点项目建设。扩大投资规模，优化投资结构，今年初步安排市重点项目190项，年度计划投资405.2亿元。下大力气抓好广东南车、台山核电、海信二期、康师傅、普利司通、星辉造纸、富华重工等重大产业项目，江顺大桥、江门大道北线、广中江高速、江罗高速、新台高速南延线、新中一级公路等重大交通基础设施项目。加快推进江门大道南线、中开高速、广佛江珠城轨前期工作，力促深茂铁路年内动工，配合做好与港珠澳大桥的规划衔接工作。加快推进高新区公共码头建设。积极落实鼓励和引导民间投资的相关政策，鼓励多种投资主体参与基础设施、市政公用等领域投资。

　　强化核心园区建设。坚持“建大平台，招优质项目”思路，大力优化园区资源配置，推动江门国家高新区体制机制创新和“一区多园”规划建设，抓好江门产业转移工业园扩园工作，加快推进江沙示范园区、轨道交通产业园、银洲湖纸业基地、台山工业新城、鹤山工业城等核心园区建设。进一步推进绿色光源、轨道交通装备、汽车零部件等省市共建产业基地建设，加快中国江门国际绿色光源博览交易中心建设。抓好高端新型电子信息、装备制造、生物医药、新光源、新能源、新材料等新兴产业发展，大力培育主导产业，构建现代产业体系。

　　强化民营经济发展帮扶。实施民营企业“双百”腾飞计划，重点扶持100家“腾飞企业”和100家“展翅企业”。构建中小微企业综合服务体系，加快中小企业窗口服务平台建设，推动各省级专业镇建设中小微企业公共服务平台。严格落实企业税费减免政策，切实减轻企业负担。支持工程（项目）采购本地企业产品，提高面向中小企业政府采购比例。鼓励发展总部经济，引导和支持非法人分支机构或三级分支机构转升为法人子公司或二级分支机构。

　　强化创新驱动。改善创新环境，提高企业自主创新能力，实施机电、纺织、食品、电子信息、造纸、建材等六大传统支柱产业转型升级行动，进一步推进“四个100”工程，推动10个产业集群转型升级[21]。扶持江门国家高新区以“三资融合”模式建设总部科技园和科技孵化器。大力支持国家级、省级企业技术中心、工程研发中心以及专业镇公共服务平台建设，组织实施100项市级以上重点科技计划项目，新培育扶持35家高新技术企业，开展产学研项目80项以上，推广科技成果500项以上，创建10家市级以上企业工程研究开发中心。大力推进“质量强市”和“品牌兴市”战略，鼓励企业争创名牌和参与国际、国家、行业标准制（修）订。

　　二、大力培育新经济增长点

　　开启海洋经济发展新引擎。加快修编《江门市海洋功能区划》和编制《江门市海岸保护与利用规划》。推进大广海湾区[22]规划建设，加强土地资源和海洋资源的保护和调控，做好交通、园区、产业、城镇等规划，争取把大广海湾区列入省级新区开发规划。加大省市共建海洋经济强市力度，提高海域和海岛管理水平，抓好“海域使用直通车”试点工作，推进集中集约用海、科学围填海和无居民海岛保护开发，广海湾争取年内处置香港惰性拆建物料1000万吨、围填海80公顷。推动崖门3万吨级航道、镇海湾3000吨级航道整治工作。培育壮大海洋新兴产业，推进临港装备制造业、港口物流、滨海旅游等产业发展。加快崖门国家一级渔港、广海国家二类渔港建设，抓好横山渔港申报国家一级渔港和沙堤渔港申报国家中心渔港工作。

　　加快以旅游、金融、物流为重点的现代服务业发展。围绕打造生态文化、滨海度假、休闲侨都三大旅游产业集聚区，大力统筹整合全市旅游资源，提升与港澳、珠三角、外省及国际旅游合作水平，加快旅游业转型升级。增强金融业服务实体经济发展能力，拓宽政银企合作平台，推进台山、开平、鹤山3家农信社改革，强化中小企业融资扶持，支持促进企业上市，发展创业投资和风险投资。支持会计、律师等专业服务行业有序发展。进一步加快银洲湖、鹤山国际物流港、南新区等现代物流基地建设。大力发展电子商务，积极培育一批电子商务示范企业。通过深化收入分配制度改革、提高城乡居民收入、稳定物价水平等方式，增强居民消费预期，提升居民消费能力，多渠道扩大消费需求。重点培育幸福导向型产业体系[23]，推广信贷、租赁等新型消费模式。继续鼓励和引导个体工商户转型升级。举办消费促进月活动，深入实施万村千乡工程[24]和家电节能惠民工程，活跃消费市场。合理引导住房消费，促进房地产业健康发展。

　　三、提高开放型经济水平

　　强化招商引资和增资扩产工作。重点瞄准世界500强、央企、大型民企、上市公司和经济中心城市，大力开展富有针对性、成效性的招商活动。加强2012年江门市招商推介会等招商活动的项目落实和信息跟踪，加快重大优质项目落户投产和建设进度。招商引资鼓励发展税收型产业和项目，开展以投资强度、亩产税收为主的项目准入考核和批后监管。搭建好乡贤回归服务平台，扎实推进“反哺工程”和“引智引制”。继续大力推动优质企业增资扩产，优化政府服务，促使企业不断发展壮大。

　　推动外贸转型升级和市场开拓。鼓励外贸企业培育自主知识产权和品牌，推动出口产品创意设计。实施进出口市场多元化战略，推进加工贸易转型升级。加强摩托车、家居用品、电子家电、水暖卫浴、箱包皮具等优势产品的市场推销。扩大国内紧缺资源、先进技术设备及与人民群众密切相关的生活用品进口。加强“大通关”建设，推进外经贸、海关、检验检疫和企业“四方联网”。支持专业市场和电子商务企业促销江门产品，举办摩托车工业博览会等展会，提高江货的市场覆盖面和占有率。

　　四、推动城乡区域协调发展

　　着力推进城镇化。优化城市功能定位，促进中心城区扩容提质，统筹加快城关镇、中心镇、特大镇发展，不断提高城市人口和产业集聚能力。大力推进投融资体制改革，整合做大投融资平台。统筹推进滨江新区建设和旧城区改造，加快江门体育中心建设，抓好广东银葵综合医院和滨江新区中小学建设，推进甘化厂片区、江会路片区、石湾直街历史文化街区“三旧”改造工作。进一步完善商业布局规划，加快推进江门国翔时代广场、益丞国际广场、金汇国际金融大厦、江海西江新城、新会美吉特广场、台山中环广场、开平东汇城、鹤山广场、恩平锦江国际新城等大型城市综合体或商贸中心建设。加快江门、新会城轨站周边地区的公交导向型土地综合开发。

　　着力抓好农村基础设施建设。加快城市基础设施向农村地区延伸和覆盖。抓好“农田水利万宗工程”和“双千”工程等民生水利工程建设，组织实施大中型灌区节水改造工程、中小河流整治工程、海堤达标加固工程等项目建设。以农村“五改”[25]和“三清”[26]工程为重点，抓好全市村庄整治工作，确保今年开展整治的村庄达710条、占全市行政村总数的68%。年内建成3个宜居城镇、14个宜居村庄、1个名镇、20个名村和一批园林镇、园林村。推进幸福安居示范村建设。

　　着力加大强农、惠农、富农力度。今年全市各级财政预算用于“三农”支出62.2亿元，比上年增长21%。继续抓好种粮直补、综合直补、农机购置补贴、农作物良种补贴等财政补贴政策，全面铺开政策性水稻种植保险工作。扶持农村集体经济发展，大力拓宽农民增收渠道，增加工资性、财产性和转移性收入。深化农村综合改革。做好省“双到”扶贫验收和新一轮启动工作。

　　着力加快现代农业发展。今年新发展市级以上农业龙头企业5家以上，发展或改造市级农民专业合作示范社65家以上。加大现代农业综合示范基地建设力度，推进开平国家现代农业示范区、粤台（江门）农业合作试验区和台山都斛万亩水稻高产示范田建设。加强农业综合开发，扎实推进新一轮现代标准农田建设。继续深化与农业部直属科研院所的合作。建立健全农业科技创新和科技推广体系。大力发展特色农产品、水产品加工业。支持农产品物流和加工体系建设，开展农超对接、农校对接、农企对接等多种形式产销对接。发展“农超对接”农民专业合作社15家，完善规范“农资放心店”30家，完善和新建农副产品连锁经营配送网点50个。落实粮食安全责任制。加大“三品一标”开发力度。

　　五、营造美丽舒适的城乡环境

　　抓好植绿建园。以创建国家生态园林城市为动力，进一步深化绿色行动，加强绿道建设，美化绿化天沙河景观带，抓好蓬江丰乐山公园、鸡爪山公园、元宝山体育公园、东湖公园桂花园和毛杜鹃园、良化西公园、江海白水带市花园和桃花园、江门国家高新区南湖项目以及新会石涧郊野公园、荔枝山公园、南湖公园、台山石花山公园、恩平锦江湿地公园等一批社区公园、城市公园、郊野公园、森林公园、体育公园建设或改造。抓好市区白石大道、双龙大道、江海路和龙湾路等道路的绿化升级改造，把城市主要道路建成绿美结合的林荫大道，市区林荫路推广率85%以上。抓好市区立体绿化建设。年内建设生态景观林带133.8公里、碳汇工程林4.56万亩。力争今年人均公园绿地面积达17平方米以上。

　　狠抓环境保护。进一步加大对自然山体和湿地绿地的保护力度。严格环保准入和污染物排放标准。实行最严格的水资源管理制度，开展饮用水源保护区综合整治，落实潭江流域水环境保护工作，在潭江流域建成2－3个村镇污水处理设施。加快城镇污水处理设施建设，年内完成杜阮污水处理厂首期、棠下污水处理厂、高新区综合污水处理厂建设，全市城镇污水处理率达到84%以上。推进广场环路、潮连嘉和污水管网及丰乐污水截污干管、江海污水处理截污管网二期工程、礼乐片区截污工程等五大污水管网工程加快建设。加大生活垃圾特别是农村生活垃圾的收集、清运力度，开展垃圾分类试点工作，推进旗杆石生活垃圾卫生填埋场二期工程建设，全市所有建制镇建成1座生活垃圾中转站，无害化处理率达90%。以控制PM2.5和臭氧等二次污染物形成为重点，实施清洁空气行动计划，大力推行机动车环保标志管理，加大黄标车[27]管理力度，加快实施中心城区黄标车限行。进一步加大重金属污染防治力度。加强环境监管，健全生态环境保护责任追究制度和环境损害赔偿制度。

　　力促资源节约。严格落实节能减排目标责任制，加强节能监察。大力推进循环经济产业园建设，推行清洁生产，发展环保产业，淘汰高能耗、高排放落后产能。大力推进省低碳城市试点建设和新会区国家可持续发展实验区建设。推广绿色建筑。加快“节水型城市”建设步伐。积极推进节约集约用地，加大力度盘活存量土地和处置闲置土地，严格执行工业项目用地准入条件。严守耕地保护红线。

　　六、提升侨乡文化软实力

　　提升公民文明道德素养。加强社会主义核心价值体系宣传教育，大力弘扬“厚于德、诚于信、敏于行”的新时期广东精神和“进取、勤劳、开放、包容”的华侨精神，形成我为人人、人人为我的良好社会风尚。加强信用城市建设，全面提升城市文明程度和市民文明素质。

　　丰富群众精神文化生活。广泛开展群众性文体活动，开展文化下乡进社区活动300场，新建或改造农村（社区）文化室308个，农村电影放映12540场，确保每月为农民和农民工放映一场电影，实现文化信息资源共享工程基层服务点覆盖每个行政村。加强文化基础设施规划建设，完成华博馆室外场景布置工作，启动江门大剧院、新图书馆、新文化馆、城市文化雕塑等标志性文化工程建设或前期工作。实现农村广播电视“户户通”。

　　加强侨乡文化保护传承。办好第五届中国(江门)侨乡华人嘉年华活动，进一步打响侨乡品牌。加大开平碉楼与村落等文化遗产、长堤历史文化街区等全市历史风貌街区和甘化厂工业遗产的保护和开发力度，深入挖掘和整理非物质文化遗产项目。创新传承曲艺、武术、古典家具及陈皮、葵艺、台山玉、龙舟、龙狮等民间特色文化。启动华博馆“网上博物馆”建设。做好“广东侨批”[28]申遗工作。

　　增强文化产业发展实力。鼓励和支持动漫等文化产品的创意设计和制作，大力发展广告、现代传媒、印刷发行、舞台美术等产业。深入开展“扫黄打非”，加强文化市场综合执法，规范文化市场秩序。办好第三届江门侨乡动漫节。

　　七、加强社会建设

　　培育发展社会组织。抓好社会组织孵化基地建设，重点推进工商经济类、公益慈善类、社会服务类、群众生活类社会组织发展。制定政府职能转移目录与政府购买服务目录，推动政府向社会组织购买服务资质评判标准化。充分发挥工会、共青团、妇联等枢纽型社会组织的作用。壮大社工服务队伍，进一步发展义工事业。

　　加强社会管理创新。加大“智慧江门”建设力度，提高城市管理和社会管理智能化、科学化水平。全面推进县、镇、村三级“社会综合服务网络”建设, 扎实推进社区基础设施“六个一”工程[29]建设，实现100%社区（村）建立公共服务站，推动家庭服务中心建设。健全基层群众自治机制。加快推进户籍行政审批制度改革，继续推动流动人口服务管理“一证通”制度落实。加强综治信访维稳网络建设，落实领导“一岗双责”和包案责任制，发挥镇（街）综治信访维稳中心作用。推广鹤山“大调解”经验，提高社会矛盾纠纷排查化解工作能力。

　　深入推进“三打两建”。进一步巩固“三打”成果，以打促建，边打边建，在抓好蓬江区、新会区、开平市等“两建”试点地区，以及信贷、纳税、建筑业和食品、农资、网吧等“两建”试点行业的基础上，全面铺开“两建”工作，优化社会信用环境和健全市场监管机制。

　　抓好平安江门建设。健全动态治安防控体系，开展社会治安重点地区集中整治，巩固平安和谐社区和民主法治村（居）创建成果。深入推进社区警务建设，加强公安派出所消防管理，大力推广网上警务室等新型警务模式。严格落实党政领导安全生产“一岗双责”、企业安全生产主体责任，坚决遏制较大以上事故发生。建立健全覆盖城乡的公共突发事件应急处置体系，建立公共突发事件预警信息发布中心，完善区域、部门联动协调机制，提高有效应对自然灾害、事故灾难以及公共卫生、食品药品安全、社会安全、环境安全事件能力和群众自救互救能力。

　　此外，继续加强外事、侨务、港澳工作，进一步推进华侨华人新生代工作。要维护妇女儿童合法权益，加快市妇女儿童活动中心筹建工作。加强国防教育，完善国防动员体系，落实双拥和优抚安置工作。继续做好民族、宗教、对台、人防、打私、统计、审计、档案、方志、气象、红十字会等工作。

　　八、进一步改善民生

　　今年各级财政预算民生投入90.9亿元，比上年增长19.19%。进一步健全“创文”长效机制，巩固“创文”成果，最大限度实现好、维护好、发展好广大人民群众的根本利益，让人民群众共享经济社会发展成果。

　　实现更高质量就业。城镇新增就业4.5万人，培训农村劳动力1.5万人，转移农村劳动力就业2万人。做好以高校毕业生为重点的青年、农村转移劳动力、城镇困难人员、退役军人就业以及失业人员再就业工作。深入推进校企合作，广泛开展“校园对接，双招互助”工作。发挥引进高层次人才“一站式”服务平台作用，优化人才发展环境。加快高技能人才实训基地建设，新增高技能人才1.2万人。加强劳动保障监察和劳动争议调解仲裁。

　　统筹城乡社会保障。抓好社会保险扩面征缴，做好城乡居民社会养老保险与城镇职工基本养老保险制度的衔接，提高城乡居民基本医疗保险补助标准。深入推进稳价惠民“三项建设”[30]。落实社会救助和保障标准与物价上涨挂钩联动机制，及时发放价格临时补贴。全市新开工保障性住房3021套，建成保障性住房4258套。完成华侨农场危房改造725户。提高社会救助水平，全市城乡低保月人均补差分别提高到242元和109元以上，确保“五保”供养标准达到上年度农村居民人均纯收入的60%以上。完善养老服务体系，积极推动民办养老机构发展，创建农村机构养老示范点，全市新增养老床位2000张。全面落实80岁以上高龄老人津贴发放工作。加快发展慈善、社会福利和残疾人事业，办好“扶贫济困日”活动。

　　坚持教育优先发展。努力办好人民满意的教育，巩固“创强”成果，加快推进教育现代化步伐，新增1个省推进教育现代化先进区。推动学前教育扩容普及和幼儿园“规范促优”。全面实施素质教育，推进中小学规范化学校建设。推动高等教育内涵式发展，办好五邑大学，加快发展适应新形势要求的现代职业教育，推进江门职业技术学院省级示范性高职院校建设，发挥高校和职业教育对江门产业发展的人才和智力支撑作用。扎实推进“强师工程”。抓好特殊学校建设。规范和促进民办教育健康发展，完善全民终身教育体系。大力保障校园、校车安全。

　　提高群众健康水平。深化医药卫生体制改革，加强全市三级医疗卫生服务体系建设，扎实推进县级公立医院综合改革试点工作，鼓励社会资本举办非公办医疗机构，加强对民办医疗机构的监管。推进基本公共卫生服务均等化，努力创建国家级、省级示范社区卫生服务中心，推进卫生城市、卫生镇、卫生村创建。开展“平价医院、平价诊室、平价药包”建设。全市新改建农村户厕1万户。年内各类食品药品生产经营单位安全信用档案建档数达7232家。加快全国“阳光家园”示范区[31]建设。积极做好第十五届省运会申办工作。广泛开展全民健身运动，进一步大力举办“百姓健康舞”、“全民健步”、“社区居民篮球赛”、“绿道体育”等健身活动。力争社区健身设施覆盖率达到90%。实施优生健康惠民工程，确保完成省下达的人口计生任务。

　　保障群众便利出行。加大公共交通投入，加强公交专用道和智能交通系统建设。更换和新增市区公交车150标台，加快市区公交站场（枢纽站）的规划建设。抓好市区主干道、高速公路出入口的建设改造以及重要地段、重点路口的改造升级，加快推进建设路——迎宾路立交、金瓯路、江睦路、胜利南路、连海北路、台山台南大道和稔广线台山段、开平环城公路、恩平环城快速路等城市路桥建设。完善城市功能布局，创新交通管理模式，着力缓解交通拥堵问题，抓好公共停车场的规划、建设和管理。加强渡口渡船管理。全市完成农村公路建设150公里。

　　九、抓好政府建设

　　做好政府工作，关键要做到求真务实、开拓进取，必须持续改进政府管理，优化政府服务，继续大力提升执行力、协同力、公信力和创新力，推进政府服务更便利、更优质、更高效、更贴心。

　　推动改革创新。坚持实事求是、解放思想，不回避问题，不回避困难，改进和创新工作方式方法，多采取现场办公会、督办会、协调会等方式，迅速解决工作中的关键性问题。坚持推进重大项目、重大活动、民生实事以及重大决策的倒逼机制，以时限、目标促使干部提高工作标准，激发干部干事创业干劲。深化重点领域改革，进一步推进大部门体制改革、简政强镇事权改革、城市基层管理扁平化改革，推动政府职能向创造良好发展环境、提供优质公共服务、维护社会公平正义转变。借鉴先进地区经验，加快推进政务服务向农村、社区延伸。加强农村“三资”管理，大力推进“两公开一监督”[32]。推进营业税改增值税试点改革。

　　推动法治建设。继续深化依法治市各项工作，推进依法行政。认真落实人大及其常委会各项决议决定，主动接受人大依法监督，支持政协委员发挥参政议政作用，提高人大代表议案、建议和政协委员提案办理质量。坚持用法规和制度管权、管事、管人，完善各项制度，最大限度减少体制和制度漏洞，提高行政运作的制度化、法治化水平。强化绩效考核、行政问责，探索推进将政府绩效管理纳入电子监察。

　　推动作风转变。严格执行中央关于改进工作作风、密切联系群众的八项规定。提高政府服务水平，加大涉企涉民部门利民便民措施的出台落实和宣传引导力度。进一步深化行政审批制度改革，各级审批事项压减率达35%左右。建立行政审批事项目录管理和公示听证制度。加强电子政务建设，进一步完善网上办事大厅建设，深入推进信息资源整合共享，拓展网上受理、并联审批和一站式服务。加快推进商事登记制度改革试点，逐步推广网上登记注册、年检。完善“12345”政府服务热线办理机制，健全行政复议、投诉申诉、网络问政等利民便民制度，畅通民意表达渠道，扩大公众对政府工作的有序参与，实现行政投诉具责受理。推进重大行政决策专家咨询论证工作，充分听取各民主党派、工商联、无党派人士和各人民团体意见，建立完善决策问责和纠错制度，推进依法决策、科学决策、民主决策。

　　推动廉政建设。大力推进廉洁城市建设，严格落实党风廉政建设责任制。严格执行公务购车和用车经费、会议经费、公务接待费用、机关出国（境）费用、办公经费预算五个“零增长”。深化政务公开，加强对政府性资金和投资项目的审计监督。继续加大案件查处力度，严惩腐败分子，以反腐倡廉的实际成效取信于民。

　　各位代表，做好新一年工作任务艰巨、使命光荣。让我们在省委、省政府和市委的正确领导下，团结带领全市人民，解放思想，改革开放，凝聚力量，攻坚克难，为建设幸福侨乡而努力奋斗！
